# Tracing evolutionary decoupling of oral and pharyngeal jaws in cichlid fishes

**DOI:** 10.1002/evl3.257

**Published:** 2021-10-05

**Authors:** Fabrizia Ronco, Walter Salzburger

**Affiliations:** ^1^ Zoological Institute, Department of Environmental Sciences University of Basel Basel CH‐4051 Switzerland

**Keywords:** Adaptive radiation, key innovation, Lake Tanganyika, morphological integration

## Abstract

Evolutionary innovations can facilitate diversification if the novel trait enables a lineage to exploit new niches or by expanding character space. The elaborate pharyngeal jaw apparatus of cichlid fishes is often referred to as an evolutionary “key innovation” that has promoted the spectacular adaptive radiations in these fishes. This goes back to the idea that the structural and functional independence of the oral and pharyngeal jaws for food capturing and food processing, respectively, permitted each jaw type to follow independent evolutionary trajectories. This “evolutionary decoupling” is thought to have facilitated novel trait combinations and, hence, ecological specialization, ultimately allowing more species to coexist in sympatry. Here, we test the hypotheses of evolutionary decoupling of the oral and pharyngeal jaws in the massive adaptive radiation of cichlid fishes in African Lake Tanganyika. Based on phylogenetic comparative analyses of oral jaw morphology and lower pharyngeal jaw shape across most of the ∼240 cichlid species occurring in that lake, we show that the two jaws evolved coupled along the main axes of morphological variation, yet most other components of these trait complexes evolved largely independently over the course of the radiation. Further, we find limited correlations between the two jaws in both overall divergence and evolutionary rates. Moreover, we show that the two jaws were evolutionary decoupled at a late stage of the radiation, suggesting that decoupling contributed to micro‐niche partitioning and the associated rapidly increasing trophic diversity during this phase.

**Impact Summary**

The East African Great Lakes Tanganyika, Malawi, and Victoria harbor extraordinarily diverse communities of cichlid fishes. Hundreds of species have evolved in each of these lakes in a relatively short period of time. The closely related cichlid species in these lakes differ greatly in size, body shape, and mouth morphology, reflecting their adaptations to various ecological niches. It has previously been suggested that the pharyngeal jaw apparatus (i.e., a second set of jaws situated in the throat of these fishes and used for food processing) has played an important role in triggering the “explosive” evolution of these fishes. It is thought that the pharyngeal jaw apparatus has freed the oral jaws from its initial dual function in food capturing and processing, so that the two types of jaws could follow different evolutionary trajectories: Although the pharyngeal jaws adapted to food processing, the oral jaws could specialize in efficient food capturing. In this study, we tested the hypothesis of “evolutionary decoupling” of the two sets of jaws across virtually all approximately 240 cichlid species occurring in Lake Tanganyika. By reconstructing the evolution of these two types of jaws throughout the phylogeny of the cichlid fishes in Lake Tanganyika, we found that the oral and pharyngeal jaws evolved nonindependently when compared across the major morphological axes. However, in most other trait axes, the two jaws showed signals of evolutionary decoupling. Further, our analyses revealed that the two jaws evolved independently over the last 2 million years, suggesting that evolutionary decoupling contributed to specialization and diversification at this later phase in the evolution of cichlid fishes in Lake Tanganyika.

Adaptive radiation refers to the rapid diversification of an organismal lineage as a result of adaptations to different ecological niches (Simpson 1953; Schluter 2000). This evolutionary process typically produces an array of ecologically and morphologically distinct species from a common ancestor in a relatively short period of time, thus being an important source of biological diversity (Schluter 2000; Gavrilets and Losos 2009). Adaptive radiation is assumed to require “ecological opportunity” that arises when an ancestral species is exposed to a novel environment with abundant and underused resources. This may happen following the colonization of a novel environment with abundant resources (e.g., a newly formed lake or island), after niche space has been freed (e.g., through extinction), or via the evolution of a novel and beneficial trait—a so‐called “key innovation”—that opens up new character space (Simpson 1953; Schluter 2000; Galis 2001).

There are three principal ecological mechanisms how a key innovation can promote diversification (Heard and Hauser 1995). The first is that the novel trait constitutes an evolutionary breakthrough in function, allowing its bearers to move into unexploited niche space. For example, the adaptive radiation of the “Antarctic clade” of notothenioid fishes in the cold waters of Antarctica has been implicated with the evolution of antifreeze glycoproteins permitting these perciform fish to thrive in areas with subzero water temperatures that are uninhabitable for competitors (Chen et al. 1997; Matschiner et al. 2011; Near et al. 2012). The second mechanism is that a key innovation substantially increases the fitness of its bearers, providing an advantage over competitors via a more efficient exploitation of resources or a more successful avoidance of predation or parasitism. For instance, latex and resin canals in various plant clades function as effective defense strategy against herbivores, promoting diversification in these clades (Farrell et al. 1991). The third ecological mechanism how a key innovation can increase diversity is through increased specialization. Conceptually, a key innovation of this kind changes the dimensionality of the morphospace by facilitating novel functional trait combinations with existing traits, thereby directly increasing ecomorphological disparity but also leading to increased specialization, which in turn allows more species to coexist (Schaefer and Lauder 1986; Heard and Hauser 1995; Wainwright 2007). In this scenario, the novel trait can only act as a key innovation if it becomes liberated from evolutionary trade‐offs, allowing it to evolve independently from other traits.

The pharyngeal jaw apparatus of members of the teleost fish families Cichlidae and Labridae ranks among the most widely cited examples of a key innovation hypothesized to promote diversification (Liem 1973; Liem and Sanderson 1986; Hulsey 2006; Wainwright 2006; Glor 2010; Wainwright et al. 2012, but see Alfaro et al. 2009). This pharyngeal trait complex, which is structurally independent of the oral jaws and developmentally derived from the fifth pair of ceratobranchials, functions as a second set of jaws (Liem 1973; Hulsey 2006) (see Fig. 1). Most teleost fishes possess toothed and versatile pharyngeal jaws that are used to process food (Lauder 1982; Galis and Drucker 1996). However, cichlids, labrids, and a few other lineages exhibit modifications in muscular sling connections and completely fused lower ceratobranchials, resulting in a greater biting force (Liem 1973; Hulsey 2006). In particular, cichlid fishes are highly diverse in the morphology of their lower pharyngeal jaw bones, reflecting a species’ adaptation to a particular feeding ecology (Barluenga et al. 2006; Muschick et al. 2012; Theis et al. 2014; Ronco et al. 2021).

**Figure 1:**
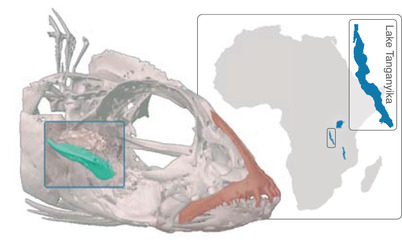
Three‐dimensional reconstruction (based on micro‐computed tomography) of the head of the cichlid species *Neolamprologus furcifer* from Lake Tanganyika (inset). The upper oral jaw (premaxilla) as part of the oral jaw apparatus is highlighted in red. A section of the skull was virtually removed (blue box) to uncover the pharyngeal jaw apparatus, with the lower pharyngeal jaw bone being highlighted in green.

In a classic article, Liem (1973) hypothesized that the presence of the pharyngeal jaw apparatus could have promoted the cichlids’ evolutionary radiations by allowing novel functional trait combinations through the evolutionary decoupling of their oral and pharyngeal jaws. This, in turn, should have minimized evolutionary trad‐offs and, hence, prevented unfavorable modifications in one of these two functional units by adaptive changes in the other. As a consequence, the two sets of jaws were permitted to follow different evolutionary trajectories: While the pharyngeal jaw apparatus could evolve toward a specialization in food processing, the oral jaws became freed from this function and could predominantly specialize in efficient food uptake. Accordingly, the evolutionary decoupling of the oral and pharyngeal jaws directly promoted ecomorphological disparity in cichlids, led to increased specialization, and enabled a more fine‐scaled niche partitioning, which in turn allowed more species to coexist.

Evolutionary decoupling is widely accepted as a potent driver of diversification (Lauder 1981; Galis 2001; Schwenk 2001; Wainwright 2007). Although the oral and pharyngeal jaws of cichlid fishes have frequently been examined in the context of feeding adaptations (e.g., Meyer 1990; Albertson et al. 2003; Muschick et al. 2011, 2012; van Rijssel et al. 2015; Ronco et al. 2021), research on the evolutionary integration of these two sets of jaws is scarce (Hulsey et al. 2006; Fraser et al. 2009; Burress et al. 2020). So far, there is evidence for evolutionary decoupling in the two types of jaws from comparative studies in Neotropical cichlids, which diversified in a complex ecological setting across the continents’ freshwater bodies (Hulsey et al. 2006; Burress et al. 2020). In contrast, the examination of Lake Malawi cichlids revealed evidence for genetic and developmental integration of the two sets of jaws (Fraser et al. 2009).

Here, we tested Liem's (1973) hypothesis of evolutionary decoupling of the oral and the pharyngeal jaws in the massive adaptive radiation of cichlid fishes in African Lake Tanganyika. This cichlid species flock comprises about 240 species that have evolved in this lake in the last 10 million years and is highly diverse in several trophic traits reflecting a wide range of feeding ecologies (e.g., Muschick et al. 2012, 2014; Ronco et al. 2019, 2021). Making use of a genome‐wide phylogeny and multivariate data on the lower pharyngeal jaw bone and the premaxilla (as part of the oral jaw apparatus) of virtually all extant cichlid species in this lake (Ronco et al. 2021), we tested for signatures of evolutionary decoupling of the two jaws by applying three complementary approaches, each targeting a different prediction related to Liem's decoupling hypothesis: (*i*) Under the assumption of coupled evolution of the jaws, one would expect that divergence in one type of jaw is tightly linked to divergence in the other. Thus, we first tested if pairwise Procrustes distances of the oral jaw correlate with pairwise distances of the lower pharyngeal jaw. (*ii*) Under the coupled‐evolution scenario, evolutionary correlations between the jaw phenotypes are expected. As a second approach, we thus tested for correlations between the three axes describing most of the phenotypic variance in each jaw type and between the axes of most morphological covariance in the two jaws. (*iii*) Under a scenario of decoupled evolution of the oral and pharyngeal jaws, evolutionary rates of the two jaws are expected to be uncorrelated. Hence, as a third approach, we tested for correlations between branch‐specific rates of morphological evolution in the two jaws across the radiation and in time slices over the phylogeny.

Overall, we found evidence for morphological integration between the major axes of shape variation, whereas most other trait components turned out to have evolved largely independently. Further, we found limited correlations between the jaws in both divergence and evolutionary rates. Finally, we found that phenotypic evolution of the two jaws was decoupled over the last 2 million years of the cichlid adaptive radiation in Lake Tanganyika, suggesting a contribution of evolutionary decoupling to the rapidly increasing trophic diversity and to micro‐niche partitioning primarily at this later phase of the radiation. Hence, all three approaches lend empirical support to Liem's hypothesis that the two jaws are—at least to some extent—evolutionary decoupled in the cichlid adaptive radiation in Lake Tanganyika.

## Methods

### DATASET

The taxon sampling of this study comprised 234 species of cichlid fishes endemic to Lake Tanganyika (Table S1), representing an almost complete sample of the cichlid fauna of this lake (183 described species plus 50 undescribed species and local variants; 91.4% of the total taxonomic diversity of cichlids in this lake). Our taxon sample covers all 12 sub‐lineages of the radiation (equivalent to the taxonomic rank of tribes) and all trophic levels (see Ronco et al. 2020, 2021).

The primary morphological data for the oral jaws consist of landmark coordinates identified on two‐dimensional X‐ray images of typically 10 specimens per species (*n* = 2171), taken from Ronco et al. (2021). Four landmarks designating the premaxilla were extracted from a set of 21 landmarks distributed over the entire skeleton (see Ronco et al. 2021 for details). The shape data for the lower pharyngeal jaw bones were based on 27 three‐dimensional landmarks derived from micro‐computed tomography scans of five specimens per species (*n* = 1154), also taken from our previous study (Ronco et al. 2021). As the landmarks were placed on the left side of the quasi‐symmetric pharyngeal jaw bone, we applied the same approach as described in Ronco et al. (2021) and mirrored the landmarks on the right side over the axis of bilateral symmetry, resulting in a dataset of 42 landmarks (see Ronco et al. 2021 for details).

Note that both morphological datasets were derived from the same formalin preserved specimens. To minimize potential artifacts due to bending of the body or head, specimens have been fixed in a standardized way (lying flat in a container with a straightened body and head; see Ronco et al. 2021 for details). To test for a potential bias in the measurements taken from two‐dimensional X‐ray images (due to the projection of a three‐dimensional structure into one focal plane), we additionally measured the length of the vertical and horizontal bone of the premaxilla in a representative subset of species with available computed tomography scans of the head in three dimensions (43 species belonging to 11 tribes). A comparison between the two‐dimensional and three‐dimensional measurements revealed high congruence between the two methods (vertical bone: *r* = 0.99, *P* < 0.0001; horizontal bone: *r* = 0.98, *P* < 0.0001; see Fig. S1)

To analyze the two morphological datasets in a phylogenetic comparative framework, we took the phylogenetic hypotheses based on genome‐wide SNPs from Ronco et al. (2021), pruned to our taxon sampling, and used species means of the phenotypic data (see below).

### MORPHOLOGICAL ALIGNMENT

All analyses presented in this study were performed in R (version 3.5.2; R Development Core Team 2018), unless specified otherwise.

As a first step, to remove information on size, position, and orientation in the morphometric data, we applied a Procrustes superimposition to the two sets of raw landmark coordinates using the R package geomorph (version 3.0.7; Adams and Otárola‐Castillo 2013). However, to obtain information on oral jaw morphology, which not only includes shape but also size and orientation of the premaxilla in relation to body size and axes, respectively, we followed the method described in Ronco et al. (2021) and extracted and re‐centered the four landmarks of the premaxilla after Procrustes superimposition of the full set of 21 landmarks distributed over the entire skeleton.

### COMPARING THE DEGREE OF DIVERGENCE BETWEEN THE TWO JAWS (APPROACH 1)

As a first approach, we tested if the degree of divergence in oral jaw morphology is associated with divergence in lower pharyngeal jaw shape. This is based on the assumption that if the two jaw types evolved completely independently, no evolutionary correlation is expected between the degree of divergence of the oral jaw morphology and divergence in pharyngeal jaw shape when taking into consideration the underlying directionality of change. To account for the full (measured) variation of the two types of jaws, we quantified divergence within each jaw using pairwise Procrustes distances in each set of multidimensional landmark data. We calculated, for each species, the mean shape of the oral jaw and the lower pharyngeal jaw bone using the function *mshape* of the R package geomorph and subsequently calculated, for each of the two jaws, all pairwise distances for all possible species comparisons using the R package Evomorph (version 0.9; Cabrera and Giri 2016). We then tested for an association between the two distance matrices, while controlling for the effect of phylogenetic distances, using a partial Mantel test as implemented in the R package vegan (version 2.5‐4; Oksanen et al. 2019). Note that, in this approach, the directionality of morphological changes is not directly evaluated, but because the underlying data are pairwise distances, species pairs with matching divergence but contradicting directionality of change will not support a correlation across species pairs.

### TESTING FOR EVOLUTIONARY CORRELATIONS BETWEEN PHENOTYPES (APPROACH 2)

The use of pairwise Procrustes distances (as described above) has the advantage that the full (measured) morphological variation can be used to test for a correlation between the divergence patterns in the two jaws. This approach indirectly accounts for the directionality of change (see above), but it does not allow to quantify the associated changes. Thus, as a second approach, we tested for an evolutionary integration along morphological trait axes between the two sets of jaws by applying two complementary strategies.

First, we tested for evolutionary correlations between the two jaws using the three axes of most variance in each type of jaw (similar to Burress et al. 2020). Although this strategy involves dimensionality reduction, it has the advantage that it permits to test for correlations between different components of each trait complex. To obtain the axes of most variance, we applied a Principal Component Analysis (PCA) to each set of Procrustes‐aligned landmarks as implemented in the R package geomorph and retained, for each dataset, the first three PC axes for further analyses. We then tested for a correlation of the first three PC axes of the oral jaw morphology with the first three PC axes of the lower pharyngeal jaw shape. To account for the phylogenetic dependence of the data, we first calculated phylogenetically independent contrasts (PICs) along the phylogeny (Felsenstein 1985) using species means of the PC scores and then calculated Pearson's *r* for PICs (R package picante, version 1.8; Kembel et al. 2010). To compare the observed effect size of evolutionary correlations with a null distribution for uncorrelated traits following a Brownian motion process, we simulated a set of 1000 traits along the phylogeny using the fastBM function of the R package phytools (version 0.6‐60; Revell 2012) and calculated evolutionary correlations among these simulated traits as described above.

As, in a PCA, the PC axes are inherently uncorrelated, we wanted to assure that the inferred evolutionary correlations between PCAs are not driven by data rotation. We therefore additionally tested for evolutionary correlations among two sets of metric trait measurements extracted from the landmark data of the two jaws. For oral jaw morphology, we used the angle of the premaxilla, the angle of the oral jaw compared to the main body axis, and the length ratio between the vertical bone and horizontal bone of the premaxilla. For the lower pharyngeal jaw bones, we extracted the length/width ratio, the horn length in relation to centroid size, the posterior thickness in relation to centroid size, and the proportion of the bone area covered with teeth.

As a second strategy to test for morphological integration—and because the PC axes and the sets of metric traits do not necessarily capture the evolutionarily correlated morphological axes—we fitted a phylogenetic two‐block partial least square model (pPLS) to the two sets of Procrustes‐aligned landmark coordinates (mean shape per species) using the phylo.integration function of the R package geomorph. This method rotates each multivariate dataset to the axis of most covariance with the other set of landmark coordinates and tests for a correlation between these two morphological axes.

### COMPARING EVOLUTIONARY RATES OF THE TWO JAWS (APPROACH 3)

As a third approach, we compared branch‐specific evolutionary rates for each jaw type across the entire radiation (conceptually following Burress et al. 2020) as well as in time‐slices over the course of the radiation (as in Cooney et al. 2017; Ronco et al. 2021). Because evolutionary rates only account for the amount of change but not for the directionality of change, only an absence of a correlation can be interpreted. This is because a correlation could reflect either evolutionary constraints (coupled evolution; elevated evolutionary rates in one jaw would directly increase evolutionary rates of the other jaw) or decoupled evolution (a scenario in which the directionality of change would need to be considered). On the other hand, under decoupled evolution, the evolutionary rates in one jaw are expected to be independent of the evolutionary rates in the other. Hence, the absence of a correlation between branch‐specific rates provides evidence for evolutionary decoupling of the two jaws (irrespective of the directionality of change), which would support Liem's evolutionary decoupling hypothesis.

To estimate rates of morphological evolution for oral and pharyngeal jaws in a Bayesian framework, we applied a multivariate variable rates model of trait evolution using the software BayesTraits (Venditti et al. 2011; http://www.evolution.rdg.ac.uk/BayesTraitsV3.0.2/BayesTraitsV3.0.2.html). This model estimates, per input variable, the ancestral state (α), a rate of evolution (σ^2^), and tests for shifts in evolutionary rates along the phylogeny across all variables. We used uniform prior distributions (α: −1 – 1, σ^2^_oral jaw_: 0 – 0.0001, σ^2^_pharyngeal jaw_: 0 – 0.001) and ran a single Markov chain Monte Carlo (MCMC) run for each jaw type (species means of PC1–PC3 of each jaw) for 1 billion iterations, sampling parameters every 100,000^th^ iteration (after a preset burnin of 10,000,000 iterations). The convergence of each chain was evaluated by calculating the effective sample size (EES) for each parameter of the posterior sample using the R package coda (version 0.19‐3; Plummer et al. 2005). As the ESS values of all parameters for oral jaw morphology exceeded 200 (see Fig. S2A), we used the posterior sample to calculate the mean relative rates per branch. For the shape of the lower pharyngeal jaw bone, the posterior sample of the evolutionary rates had a bimodal distribution for all three PC axes, whereas all other parameters converged (see Fig. S2B). To test if this bimodality indicates two distinct but equally likely optima or is because the chain had not yet converged, we run 10 additional MCMC runs with the same parameters and five longer runs (1.5 billion iterations). Because all additional 15 runs converged at the same two optima as the initial run (see Fig. S2C), we proceeded with the initial posterior sample. As the three rates were highly correlated across the chain, we divided the posterior sample into two groups according to the two optima (optimum 1: low rates for all three axes [σ^2^_PC1_ < 0.0004, σ^2^_PC2_ < 0.00023, σ^2^_PC3_ < 0.00016]; optimum 2: high rates for all three axes [σ^2^_PC1_ ≥ 0.0004, σ^2^_PC2_ ≥ 0.00023, σ^2^_PC3_ ≥ 0.00016]). We then calculated for each of these “sub‐chains” ESS values for each parameter, which then exceeded 200 in all cases. We thus summarized the posterior sample by calculating mean rates of evolution per branch from parameters of each of these two sub‐chains separately. Based on the posterior probabilities (PP) of the two optima, we selected the means of optimum 2 (high rates) for all downstream analyses (PP_optimum 1_ = 0.36, PP_optimum 2_ = 0.62). However, to verify the results, we repeated the downstream analyses using the mean rates of optimum 1 plus using 1000 random samples from the entire posterior distribution instead of mean values (Figs. S3 and S4). Further, comparing the absolute rates of evolution per branch of the two sub‐chains (evolutionary rate × branch‐specific relative rate) confirmed that the two optima in evolutionary rates inferred highly congruent rates per branch (low rates scaled with high relative rates vs. high rates scaled with low relative rates; Figs. S3A and S3B).

To test for an overall correlation between evolutionary rates, we calculated Pearson's *r* for the inferred branch‐specific evolutionary rates of the two jaws. To identify potential time periods during which the two jaws evolved decoupled, we calculated evolutionary rate correlations in sliding windows along the phylogeny. Similar to previous studies (Cooney et al. 2017; Ronco et al. 2021), we sampled evolutionary rates along the phylogeny, in this case using time windows of 0.15 million years (as in Ronco et al. 2021), and then quantified correlations of evolutionary rates through time by calculating Pearson's *r* based on the branches existing within each window (note that some branches span more than one window). Because only very few lineages existed near the onset of the radiation, we assigned all branches that existed before 8 million years ago to the first window. This resulted in a sample size of at least seven branches per window.

## Results

### COMPARING THE DEGREE OF DIVERGENCE BETWEEN THE TWO JAWS (APPROACH 1)

The comparison between pairwise Procrustes distances in oral jaw morphology and in lower pharyngeal jaw shape across the cichlid adaptive radiation in Lake Tanganyika by means of a partial Mantel test revealed a very weak, yet significant, correlation between the two distance matrices (Mantel *r* = 0.09, *P* = 0.02). The graphical representation of pairwise Procrustes distances between all pairs of species (Fig. S5) highlights the overall weak correlation between morphological divergence in the two jaw types and a largely unstructured matrix of pairwise distances. In some of the cichlid tribes of Lake Tanganyika, the mismatch between the two distance matrices was particularly pronounced. Species of the tribe Bathybatini, for example, featured among the most highly diverged lower pharyngeal jaws compared to all other species, whereas they showed little divergence in oral jaw morphology in the respective species comparisons. Likewise, some of the Tropheini showed among the largest Procrustes distances to the majority of species in oral jaw morphology, yet rather small distances in lower pharyngeal jaw shape. In contrast, the Cyprichromini showed congruent patterns of Procrustes distances between the jaw types. This indicates that, based on pairwise distances (which indirectly account for the underling directionality of change), divergence in one jaw system is only weakly connected to divergence in the other.

### EVOLUTIONARY CORRELATIONS (APPROACH 2)

**Figure 2:**
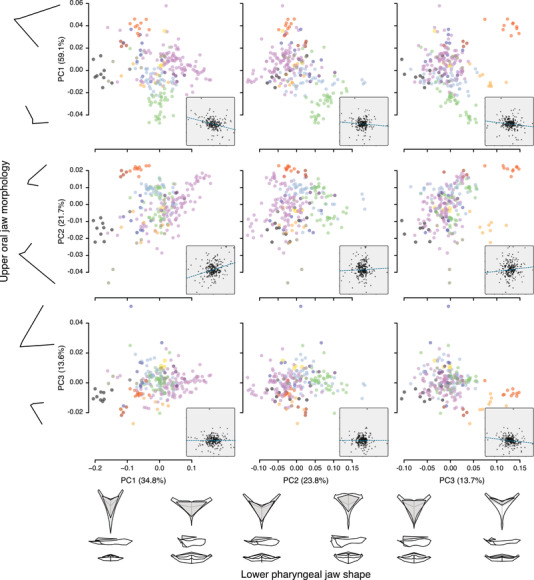
Correlations among principal components (PC1–PC3) of lower pharyngeal jaw shape (*x*‐axes) and oral jaw morphology (*y*‐axes). Data points are species means colored according to tribe (see Fig. 3). For each PC axis, the percentage of explained variance is provided in brackets and the associated shape changes (minimum and maximum) are shown. The insets display the evolutionary correlations of phylogenetically independent contrasts calculated for the PC scores (see Fig. S6 for fully sized plots).

To quantify trait combinations in oral and pharyngeal jaws across the Tanganyikan cichlid radiation, we drew on the PC analyses of Ronco et al. (2021), but instead of focusing on two‐dimensional morphospace occupation within each jaw, we compared the first three PC axes between the two jaws and calculated evolutionary correlations (Figs. 2 and S6). The re‐analyses of the landmark data, which in our case only included species for which data on both the oral and the pharyngeal jaw were available, revealed that PC1 of oral jaw morphology (explaining 59.1% of the total variance) was mainly associated with the relative size of the premaxilla and its orientation relative to the body axes. PC2 of oral jaw morphology (explaining 21.7% of the total variance) involved changes in the mouth position in combination with changes in the relative length of the lateral extensions of the bone. Similar to PC1, the underlying shape changes of PC3 of oral jaw morphology (explaining 13.6% of the total variance) involved the relative size of the premaxilla and its orientation relative to the body axes, but in this case in the opposite combinations of the two traits (small premaxilla paired with a superior mouth). For lower pharyngeal jaw shape, PC1 (explaining 34.8% of the total variance) involved changes in the anterior‐posterior elongation of the bone in relation to the two other dimensions. In contrast, PC2 of lower pharyngeal jaw shape (explaining 23.8% of the total variance) represented shifts in the length of the horns (lateral muscle attachments) and in thickness of the posterior part of the jaw. PC3 of lower pharyngeal jaw shape (explaining 13.7% of the total variance) was mainly associated with changes in the area of dentition paired with changes in (dorsoventral) thickness of the bone.

**Figure 3:**
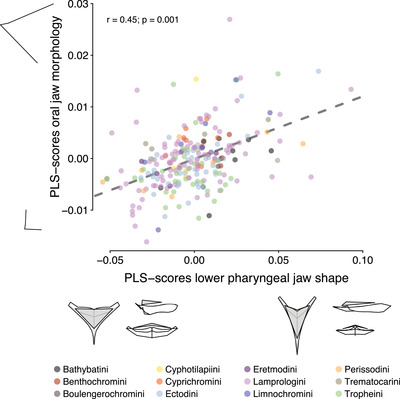
The phylogenetic two‐block partial least square (PLS) analysis of the lower pharyngeal jaw shape and oral jaw morphology revealed the axes of most covariance between the two types of jaws, which are largely congruent with PC1 of each jaw type (see Fig. 2). Data points are species means colored according to tribe and for each PLS axis, the associated shape changes (minimum and maximum) are shown.

Our analysis of evolutionary correlations revealed that PC1 of lower pharyngeal jaw shape correlated with PC1 (*r* = −0.32, *P* < 0.001, *df* = 232) and PC2 (*r* = 0.29, *P* < 0.001, *df* = 232) of oral jaw morphology. These evolutionary correlations were higher than expected under uncorrelated trait evolution (*r* = −0.21 to 0.23). This was further supported by the pPLS analysis, which identified axes of most covariance between the jaws that are very similar to PC1 of each jaw and show significant morphological integration (*r* = 0.45, *P* = 0.001; Fig. 3). Thus, a large premaxilla with a superior mouth position was evolutionary correlated with an elongated and delicate lower pharyngeal jaw bone. Focusing on the larger tribes separately, we confirmed the correlation between the first PC axes of the two jaws for Lamprologini and Ectodini (Ectodini: *r* = −0.59, *P* < 0.001; Lamprologini: *r* = −0.36, *P* < 0.001). The Tropheini on the other hand deviated from the morphological integration found across all taxa, showing substantial variation in PC1 of the oral jaw but similarity in PC1 of the lower pharyngeal jaw shape (*r* = −0.20, *P* = 0.22; Fig. 2, light green).

The opposite trait combination within the oral jaw (PC3: small premaxilla paired with a superior mouth) did not correlate with any of the trait axes of the lower pharyngeal jaw shape (all correlation coefficients fell way within the null distribution of evolutionary correlations of uncorrelated traits: PC1: *r* = 0.002, *P* = 0.97, *df* = 232; PC2: *r* = −0.002, *P* = 0.98, *df* = 232; PC3: *r* = −0.14, *P* = 0.03, *df* = 232). Likewise, PC2 of lower pharyngeal jaw shape did not correlate with any of the three PC axes of oral jaw morphology (PC1: *r* = −0.09, *P* = 0.19, *df* = 232; PC2: *r* = 0.04, *P* = 0.53, *df* = 232; PC3: *r* = −0.002, *P* = 0.97, *df* = 232). Thus, the shape axis that involves the length of the lateral muscle attachments and that scales negatively with the thickness of the posterior part of the jaw appears to be evolutionary decoupled from the oral jaw morphology in Lake Tanganyika cichlids. For PC3 of lower pharyngeal jaw shape, we found weak correlations with the three PC axes of oral jaw morphology (PC1: *r* = −0.13, *P* = 0.04, *df* = 232; PC2: *r* = 0.10, *P* = 0.13, *df* = 232; PC3: *r* = −0.14, *P* = 0.03, *df* = 232), which fell within the expectation of uncorrelated traits, suggesting an evolutionary decoupling of the area of dentition of the lower pharyngeal jaw and oral jaw morphology.

The evolutionary correlations among the meristic trait measurements extracted from the landmark data all fell within the null distribution for uncorrelated traits simulated under Brownian motion (Fig. S7).

### TEMPORAL PATTERNS OF EVOLUTIONARY RATE CORRELATIONS (APPROACH 3)

**Figure 4:**
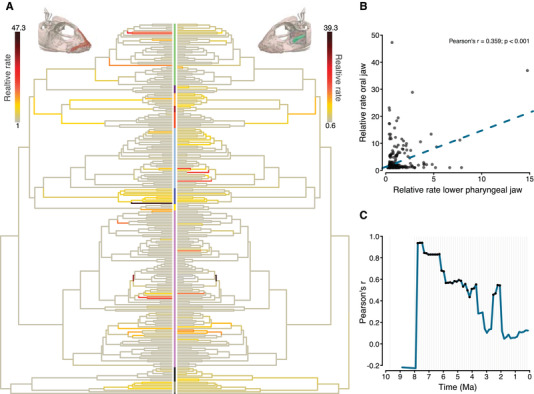
(A) Relative evolutionary rates of the first three PC axes of oral jaw morphology (left panel) and lower pharyngeal jaw shape (right panel) over the course of the adaptive radiation of cichlid fishes in Lake Tanganyika. Colors at the tips of the phylogenies correspond to the tribes (see Fig. S3). (B) Correlation of branch‐specific relative rates between the two jaws across the radiation. (C) Correlation coefficients of relative evolutionary rates between the two jaws through time (see also Figs. S3 and S4). Black dots indicate correlation coefficients that have an associated *P*‐value below 0.05.

The inferred branch‐specific evolutionary rates of jaw evolution in the cichlid adaptive radiation of Lake Tanganyika varied substantially (Fig. 4A), with a maximum relative rate of 47.3 for oral jaw morphology and maximum of a 39.3‐fold rate for lower pharyngeal jaw shape. The branch‐specific rates showed a correlation between the two jaws (Pearson's *r* = 0.36, *P* < 0.001). However, the correlation coefficient appeared to be inflated by a single branch (within the Lamprologini) with high relative rates in both sets of jaws (Figs. 4A, 4B, and S3; when this data point is excluded *r* = 0.19, *P* < 0.001). Apart from this overall significant correlation, numerous branches with low rates of lower pharyngeal jaw evolution substantially varied in rates of oral jaw evolution and vice versa. This fan‐shaped data distribution suggests that evolutionary rates of the two jaws were not strongly coupled over the course of the radiation.

When quantifying the correlations between the branch‐specific rates of the two jaws in time slices along the phylogeny, we found that the correlations varied extensively over time (Figs. 4C, S3, and S4). In the early phase of the radiation, the correlation between evolutionary rates of the two jaw types was initially high but decreased over time. However, most of the strong correlations were mainly driven by only a few branches in the phylogeny. For example, the increased correlation observed around 2–3 million years ago was again mainly driven by the one branch within the Lamprologini with high relative rates for both sets of jaws (Figs. 4 and S4). Overall, the correlations within the time slices showed a similar pattern as the one observed across all branches, namely, that lower rates in oral jaw evolution were paired with varying rates in pharyngeal jaw evolution and vice versa. Yet, this pattern became stronger over the course of the radiation, as reflected by decreasing correlation coefficients over time (Figs. 4, S3, and S4).

## Discussion

In this study, we examined the ecologically and morphologically diverse species flock of cichlid fishes in Lake Tanganyika, to test Karel Liem's (1973) hypothesis that the oral and the pharyngeal jaws are evolutionary decoupled. More precisely, Liem (1973) proposed that the evolutionary decoupling of the elaborate pharyngeal jaw apparatus and the oral jaws, as structurally and functionally independent units, facilitated independent adaptations of the oral jaws to specialize toward food capturing and of the pharyngeal jaws toward food processing.

The oral and pharyngeal jaws of cichlids have previously been identified as functionally independent units involved in trophic adaptations (Liem 1973; Galis and Drucker 1996; Hulsey and Garcia De Leon 2005). For example, it has been shown that elongated and rather delicate pharyngeal jaws are generally associated with a carnivore feeding ecology (piscivores and zooplankton feeders), whereas a wide and robust lower pharyngeal jaw is generally associated with algae grinding or mollusk crushing (Muschick et al. 2012; Ronco et al. 2021). Likewise, the oral jaw seems to be mainly associated with food uptake, whereby species feeding from the open water column have a larger premaxilla paired with a more superior mouth position and benthic feeders tend to have small premaxilla and an inferior mouth position (Hulsey and Garcia De Leon 2005; Ronco et al. 2021). In the present study, these broad‐scale trophic adaptations were captured by the first PC axes of each jaw type (Fig. 2) and by the PLS axes of most covariation (Fig. 3). Because, for each jaw type, this primary trait axes (PC1 and PLS, respectively) correlate with a very similar ecological trajectory (piscivores and zooplankton feeders eat mainly from the open water column, whereas algae grinding or mollusk crushing species feed predominantly from the benthos), it is not surprising that the direct comparison of the two trait axes revealed more evolutionary integration than expected for uncorrelated traits—either reflecting morphological integration along these particular shape axes or largely correlated selection along these broad ecological trajectories. This is in line with the overall integration of the two jaws across trophic guilds in Neotropical cichlids (Burress et al. 2020). However, even along these major trait axes some cichlid tribes in Lake Tanganyika show signatures of evolutionary decoupling. The Tropheini, for example, display very little variation along PC1 of lower pharyngeal jaw shape as opposed to substantial variation in the orientation and size of the premaxilla (Fig. 2). This suggests that the members of the Tropheini are similar in *what* they eat—they mostly process algae and cyanobacteria (Muschick et al. 2012; Tada et al. 2017) using similarly shaped pharyngeal jaws—but differ greatly in *how* they graze or browse this “aufwuchs” with varying oral jaws. Perhaps it is this micro‐niche partitioning with respect to food uptake that has facilitated co‐existence of various Tropheini species in sympatry.

Besides the overall morphological integration, most other pairwise comparisons of the PC axes showed little correlation but a broad spectrum of trait combinations (Fig. 2), suggesting that these additional trait components of the two jaw types were largely evolutionary decoupled in the cichlid adaptive radiation in Lake Tanganyika. Importantly, PC2 of lower pharyngeal jaw shape, which captures shape changes that are known to be ecologically important (Muschick et al. 2012; Ronco et al. 2021), showed no correlation with any of the PC axes of oral jaw morphology. Hence, our findings support the idea that the decoupling of certain, ecologically relevant, trait components of the oral and pharyngeal jaws in cichlids increased the dimensionality of their ecomorphospace, which could not have been exploited under the dominance of evolutionary constraints (Wainwright 2007).

The comparison of pairwise Procrustes distances between the two jaws—thus, considering the entire measured morphological variation—revealed that divergence in one jaw was only weakly correlated with divergence in the other (Fig. S5). This weak correlation in overall divergence is further supported by the inferred evolutionary rates across the first three PC axes for each jaw (Fig. 4). Apart from an overall correlation in evolutionary rates, we found a fan‐shaped data distribution (branches with similar rates in one jaw substantially varied in rates of the other jaw), which suggests that evolutionary rates of the two jaws were not strongly coupled over the course of the radiation. This limited correlation in evolutionary rates again lends support for Liem's hypothesis of an evolutionary decoupling of the two sets of jaws in Lake Tanganyika cichlids.

The role of limited evolutionary integration of the two jaw types became particularly evident when considering the temporal dynamics of evolutionary rates correlations over the course of the radiation (Figs. 4 and S4). The rates of oral and pharyngeal jaw evolution were more strongly correlated near the onset of the radiation, which is when the adaptive radiation was mainly driven by divergence in macrohabitat use (Ronco et al. 2021). This positive correlation could potentially reflect coupled evolution of the two jaws during this time period (unless the correlated rates led to uncorrelated phenotypes). Nevertheless, by sampling evolutionary rates correlations through time, we identified a time period during which the rates of the two jaws were completely uncorrelated, suggesting evolutionary decoupling at least during these last 2 million years of evolution. Importantly, this late phase of the radiation was shown to be predominantly driven by trophic divergence (Ronco et al. 2021). The temporal alignment of the absence of a correlation between evolutionary rates of the jaws and species accumulation as well as trophic divergence suggests that evolutionary decoupling—and, hence, the release of potential evolutionary constraints—might have been important relatively late in the radiation (Fig. 4C), at a time when niche space is likely to have become more and more limited.

Taken together, based on comparative analyses of oral jaw morphology and lower pharyngeal jaw shape across nearly the entire cichlid fauna of Lake Tanganyika, we show that the two jaw types show morphological integration among the major trophic axes. At the same time, we provide empirical evidence for Liem's (1973) hypothesis that the pharyngeal jaw apparatus evolved to some extent decoupled from the oral jaws. The signature of decoupled evolution in some trait components together with a very weak correlation of jaw divergence corroborates the view that the pharyngeal jaw apparatus of cichlids has acted as a key innovation by changing the dimensionality of the morphospace, thereby allowing novel trait combinations. Further, we show that the evolution of the two jaws was decoupled during the late phase of the radiation, suggesting that evolutionary decoupling promoted micro‐niche partitioning in the course of the radiation, thereby facilitating the co‐existence of multiple species in sympatry. We thus found evidence that a main ecological mechanism by which the pharyngeal jaw apparatus has promoted diversification in the cichlid adaptive radiation of Lake Tanganyika is by expanding ecomorphological disparity. In addition, the functional morphology of the pharyngeal jaw apparatus is likely to have equipped cichlids with a more efficient machinery for food processing compared to competing species from other fish families (Liem 1973; Galis and Drucker 1996; but see McGee et al. 2015) and to have made unexploited niche space accessible by increasing the repertoire of possible food recourses (e.g., mollusk crushing [Wainwright 1987; Hulsey 2006]). It thus appears that, in the case of the pharyngeal jaw apparatus of cichlid fishes, several ecological mechanisms of how a key innovation can promote diversification have acted complementary.

## Conflict of Interest

The authors declare no conflict of interest.

## Author Contributions

FR and WS conceived the study and wrote the Manuscript. FR analyzed and visualized the data.

## Data Archiving

No new data were generated for this study.

## Supporting information

Figure S1. The comparison between the length measurements based on two‐dimensional and three‐dimensional landmark coordinates of the vertical (A) and horizontal bone (B) of the premaxilla revealed high congruence between the two methods (*n* = 43).
Figure S2. Posterior sample of parameters estimated with a variable rates model of trait evolution using BayesTraits for oral jaw morphology (A) and lower pharyngeal jaw shape (B).
Figure S3. (A) Comparison of mean branch‐specific relative evolutionary rates for lower pharyngeal jaw shape between the two optima in parameters of the posterior sample (see Fig. S2).
Figure S4. Correlations of relative evolutionary rates through time based on optimum 1 (left panel) and optimum 2 (right panel) in parameters of the posterior sample.
Figure S5. Pairwise distances matrix within oral jaw morphology (upper triangle) and within lower pharyngeal jaw shape (lower triangle) of cichlid fishes in Lake Tanganyika.
Figure S6. Evolutionary correlations of the first three PC axes of oral jaw morphology (*y*‐axes) and lower pharyngeal jaw shape (*x*‐axes).
Figure S7. Evolutionary correlations of metric measurements of oral (*y*‐axes) and lower pharyngeal (*x*‐axes) jaws.
Table S1. Sample size information of the studied species.
