# Supplementary material for: Tracing evolutionary decoupling of oral and pharyngeal jaws in cichlid fishes
Source: Evol Lett. 2021 Oct 5;5(6):625–35. doi: 10.1002/evl3.257 (PMC8645199; doi:10.1002/evl3.257)
Supplement: Supplementary file 1 — Figure S1. The comparison between the length measurements based on two‐dimensional and three‐dimensional landmark coordinates of the vertical (A) and horizontal bone (B) of the premaxilla revealed high congruence between the two methods (n = 43). Figure S2. Posterior sample of parameters estimated with a variable rates model of trait evolution using BayesTraits for oral jaw morphology (A) and lower pharyngeal jaw shape (B). Figure S3. (A) Comparison of mean branch‐specific relative evolutionary rates for lower pharyngeal jaw shape between the two optima in parameters of the posterior sample (see Fig. S2). Figure S4. Correlations of relative evolutionary rates through time based on optimum 1 (left panel) and optimum 2 (right panel) in parameters of the posterior sample. Figure S5. Pairwise distances matrix within oral jaw morphology (upper triangle) and within lower pharyngeal jaw shape (lower triangle) of cichlid fishes in Lake Tanganyika. Figure S6. Evolutionary correlations of the first three PC axes of oral jaw morphology (y‐axes) and lower pharyngeal jaw shape (x‐axes). Figure S7. Evolutionary correlations of metric measurements of oral (y‐axes) and lower pharyngeal (x‐axes) jaws. Table S1. Sample size information of the studied species. [file EVL3-5-625-s001.pdf]

Supporting Information

Tracing evolutionary decoupling of oral and pharyngeal jaws in cichlid fishes

Fabrizia Ronco[1] and Walter Salzburger[1]

[1]Zoological Institute, Department of Environmental Sciences, University of Basel, Basel, Switzerland
e-mail: fabrizia.ronco@unibas.ch, walter.salzburger@unibas.ch

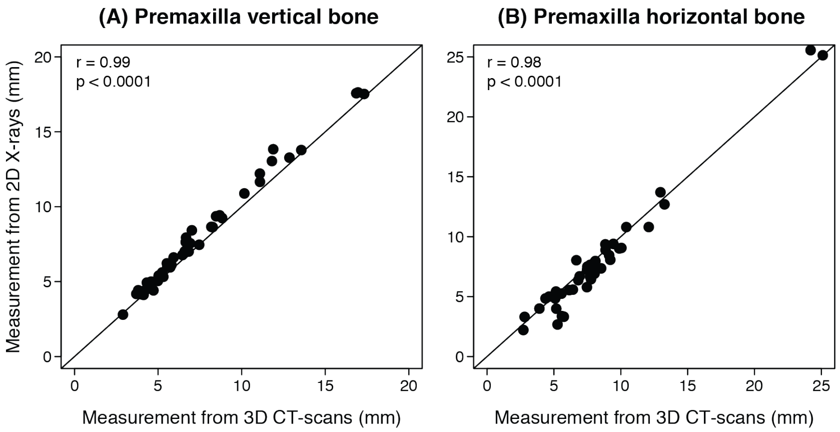

Figure S1. The comparison between the length measurements based on 2D and 3D landmark coordinates of the vertical (A) and horizontal bone (B) of the premaxilla revealed high congruence between the two methods (n = 43).

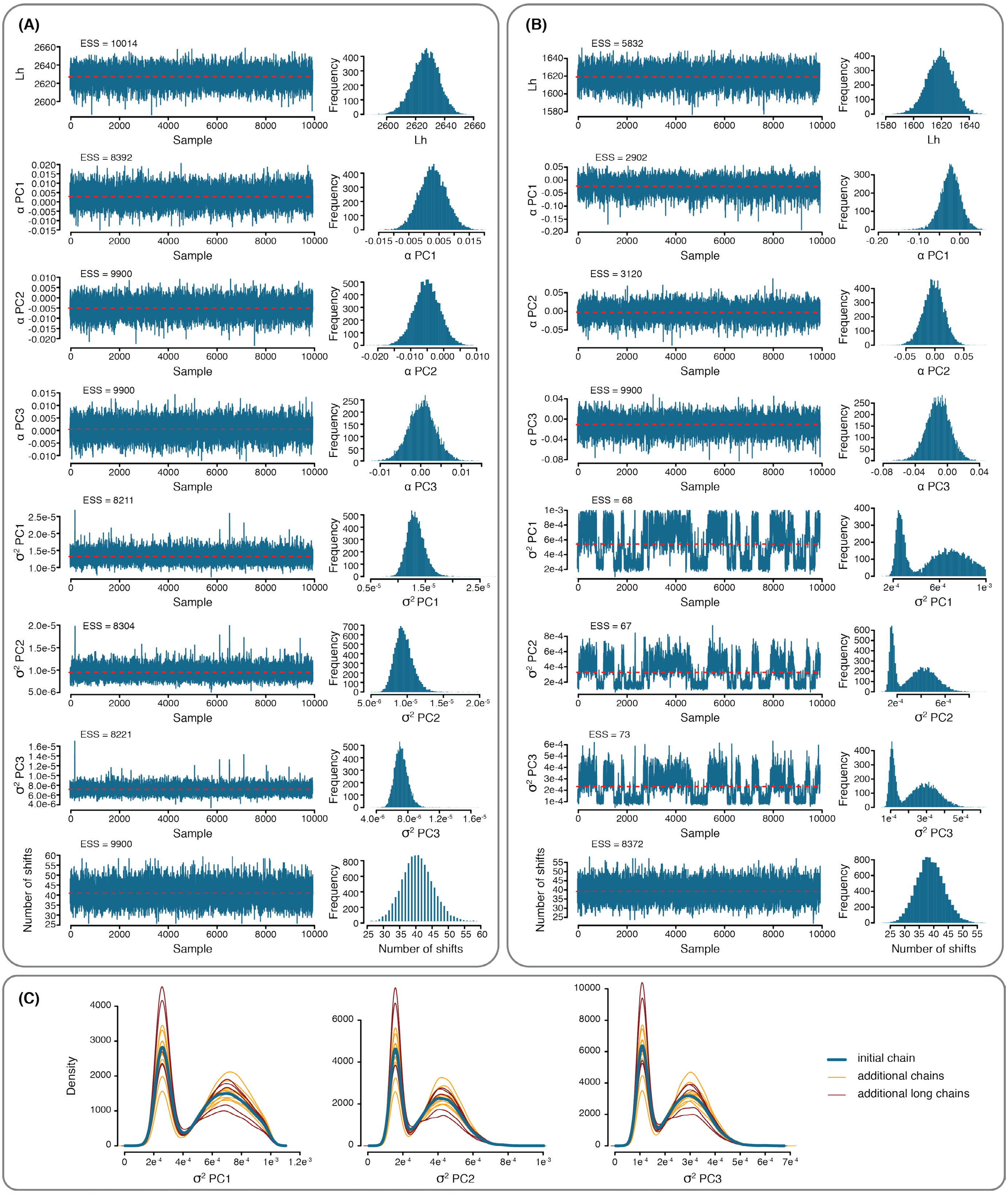

Figure S2. Posterior sample of parameters estimated with a variable rates model of trait evolution using BayesTraits for oral jaw morphology (A) and lower pharyngeal jaw shape (B). The effective sample size (ESS) for each parameter is given on top of each plot. For lower pharyngeal jaw shape the model revealed two equally likely optima of evolutionary rates (σ^2) for all three PC-axes. After splitting the posterior sample into two sub-chains based on these two optima all parameters reached convergence (see methods for details). (C) Posterior distribution of the inferred evolutionary rates for lower pharyngeal jaw shape sampled from 10 additional MCMC runs (1 billion iterations) and 5 longer MCMC runs (1.5 billion iterations). All additional runs resulted in highly congruent parameter estimates, confirming convergence at two equally likely optima of evolutionary rates (σ^2) for all three PC-axes.

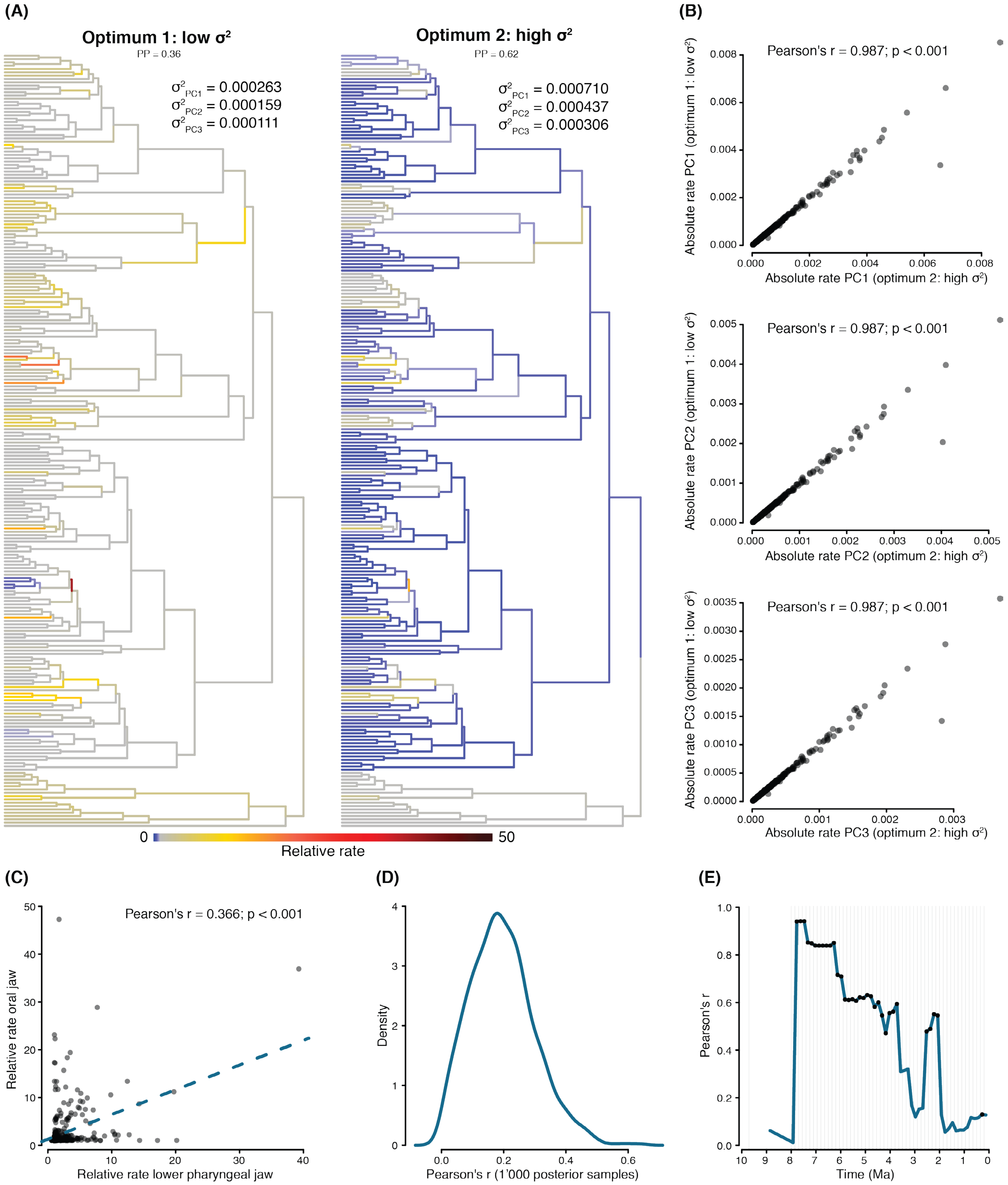

Figure S3. (A) Comparison of mean branch-specific relative evolutionary rates for lower pharyngeal jaw shape between the two optima in parameters of the posterior sample (see Fig. S2). Optimum 1 was characterized by lower overall evolutionary rates (σ^2) paired with high relative rates, while optimum 2 revealed high evolutionary rates with rate shifts mainly below 1 (PP: posterior probability). (B) Correlations between branch-specific absolute rates of lower pharyngeal jaw shape evolution (PC1–PC3) based on the two optima. For all three PC-axes the two sets of parameters resulted in highly congruent absolute rates of evolution. (C) Correlation of branch-specific relative rates of evolution between oral jaw morphology and lower pharyngeal jaw shape based on optimum 1 in parameters of the posterior sample (see also Fig. 4B). (D) Distribution of correlations of branch-specific relative rates of evolution between the two jaws based on 1'000 random samples from the entire posterior distribution. (E) Correlations of relative evolutionary rates through time based on optimum 1 in parameters of the posterior sample (see also Fig. 4C). Black dots indicate correlation coefficients that have an associated p-value below 0.05.

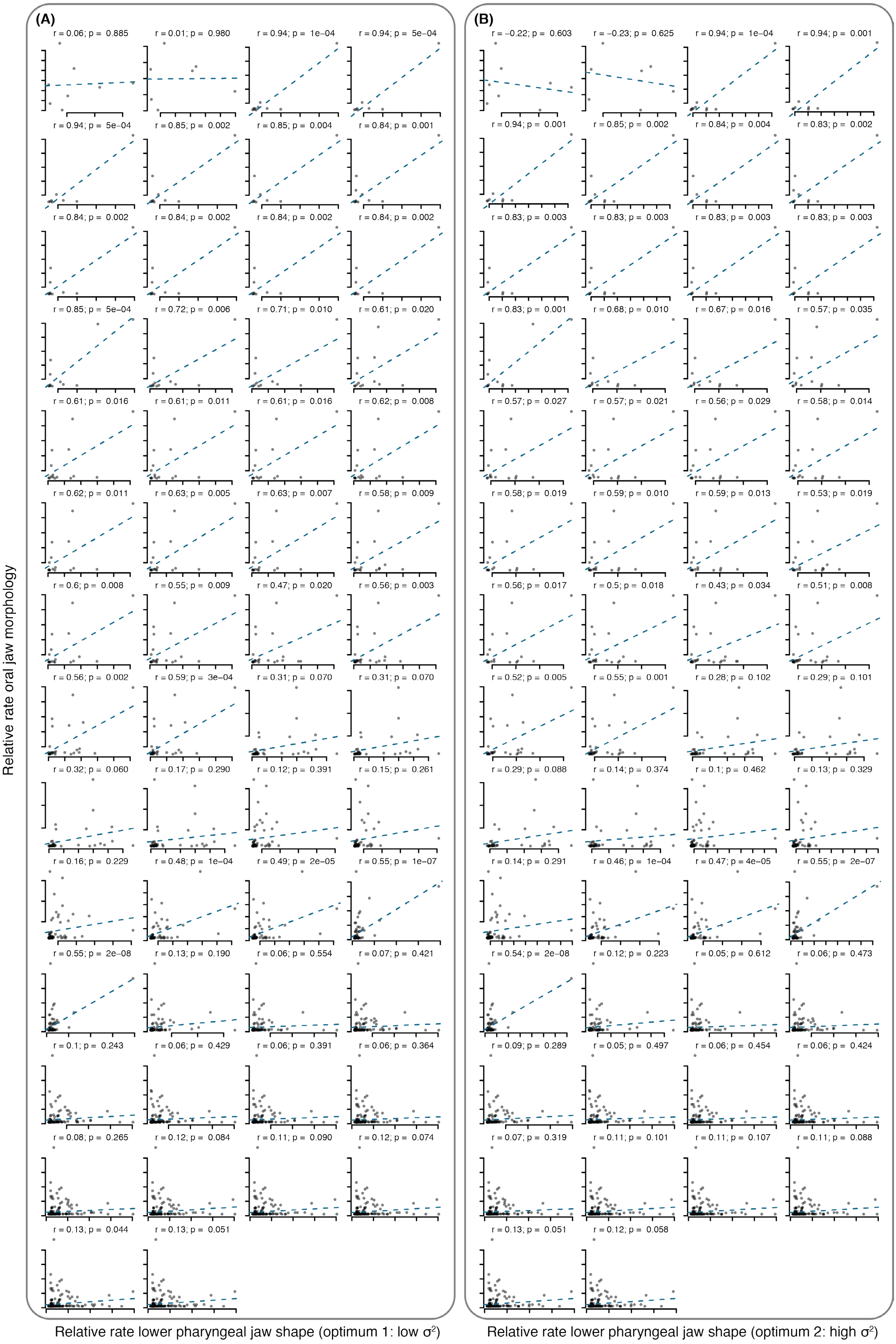

(A)
r = 0.06; p = 0.885
r = 0.01; p = 0.980
r = 0.94; p = 1e−04
r = 0.94; p = 5e−04
r = 0.94; p = 5e−04
r = 0.85; p = 0.002
r = 0.85; p = 0.004
r = 0.84; p = 0.001
r = 0.84; p = 0.002
r = 0.84; p = 0.002
r = 0.84; p = 0.002
r = 0.84; p = 0.002
r = 0.85; p = 5e−04
r = 0.72; p = 0.006
r = 0.71; p = 0.010
r = 0.61; p = 0.020
r = 0.61; p = 0.016
r = 0.61; p = 0.011
r = 0.61; p = 0.016
r = 0.62; p = 0.008
r = 0.62; p = 0.011
r = 0.63; p = 0.005
r = 0.63; p = 0.007
r = 0.58; p = 0.009
r = 0.6; p = 0.008
r = 0.55; p = 0.009
r = 0.47; p = 0.020
r = 0.56; p = 0.003
r = 0.56; p = 0.002
r = 0.59; p = 3e−04
r = 0.31; p = 0.070
r = 0.31; p = 0.070
r = 0.32; p = 0.060
r = 0.17; p = 0.290
r = 0.12; p = 0.391
r = 0.15; p = 0.261
r = 0.16; p = 0.229
r = 0.48; p = 1e−04
r = 0.49; p = 2e−05
r = 0.55; p = 1e−07
r = 0.55; p = 2e−08
r = 0.13; p = 0.190
r = 0.06; p = 0.554
r = 0.07; p = 0.421
r = 0.1; p = 0.243
r = 0.06; p = 0.429
r = 0.06; p = 0.391
r = 0.06; p = 0.364
r = 0.08; p = 0.265
r = 0.12; p = 0.084
r = 0.11; p = 0.090
r = 0.12; p = 0.074
r = 0.13; p = 0.044
r = 0.13; p = 0.051
(B)
r = −0.22; p = 0.603
r = −0.23; p = 0.625
r = 0.94; p = 1e−04
r = 0.94; p = 0.001
r = 0.94; p = 0.001
r = 0.85; p = 0.002
r = 0.84; p = 0.004
r = 0.83; p = 0.002
r = 0.83; p = 0.003
r = 0.83; p = 0.003
r = 0.83; p = 0.003
r = 0.83; p = 0.003
r = 0.83; p = 0.001
r = 0.68; p = 0.010
r = 0.67; p = 0.016
r = 0.57; p = 0.035
r = 0.57; p = 0.027
r = 0.57; p = 0.021
r = 0.56; p = 0.029
r = 0.58; p = 0.014
r = 0.58; p = 0.019
r = 0.59; p = 0.010
r = 0.59; p = 0.013
r = 0.53; p = 0.019
r = 0.56; p = 0.017
r = 0.5; p = 0.018
r = 0.43; p = 0.034
r = 0.51; p = 0.008
r = 0.52; p = 0.005
r = 0.55; p = 0.001
r = 0.28; p = 0.102
r = 0.29; p = 0.101
r = 0.29; p = 0.088
r = 0.14; p = 0.374
r = 0.1; p = 0.462
r = 0.13; p = 0.329
r = 0.14; p = 0.291
r = 0.46; p = 1e−04
r = 0.47; p = 4e−05
r = 0.55; p = 2e−07
r = 0.54; p = 2e−08
r = 0.12; p = 0.223
r = 0.05; p = 0.612
r = 0.06; p = 0.473
r = 0.09; p = 0.289
r = 0.05; p = 0.497
r = 0.06; p = 0.454
r = 0.06; p = 0.424
r = 0.07; p = 0.319
r = 0.11; p = 0.101
r = 0.11; p = 0.107
r = 0.11; p = 0.088
r = 0.13; p = 0.051
r = 0.12; p = 0.058
Relative rate oral jaw morphology
Relative rate lower pharyngeal jaw shape (optimum 1: low σ²)
Relative rate lower pharyngeal jaw shape (optimum 2: high σ²)

Figure S4. Correlations of relative evolutionary rates through time based on optimum 1 (left panel) and optimum 2 (right panel) in parameters of the posterior sample. Each plot shows the correlation of evolutionary rates in a sampled time window from past (top left) to present (bottom right).

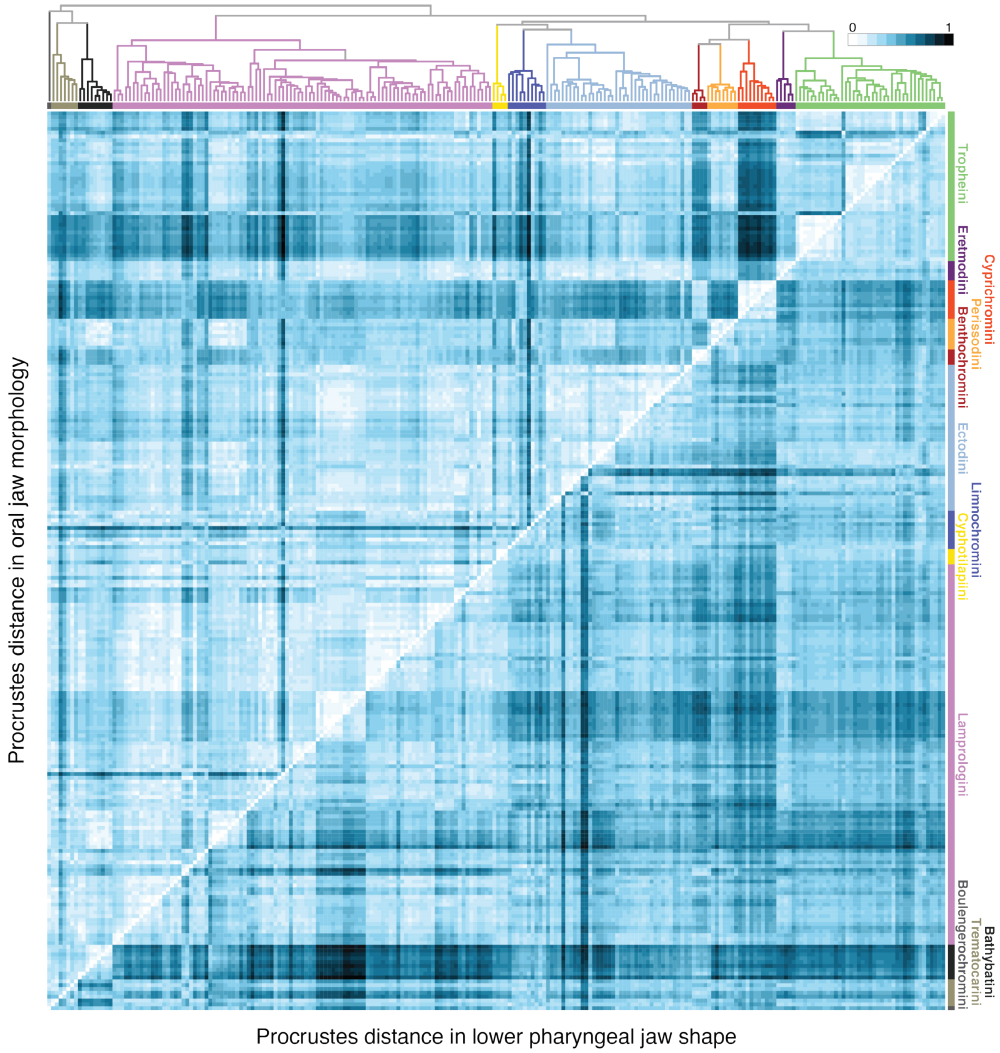

Figure S5. Pairwise distances matrix within oral jaw morphology (upper triangle) and within lower pharyngeal jaw shape (lower triangle) of cichlid fishes in Lake Tanganyika. The largely asymmetric matrix illustrates the overall weak correlation between the relative divergence of each jaw type (partial test: $r = 0.09$, $p = 0.02$). The phylogenetic relationship among the species is shown at the top of the matrix, with the branches being colored according to tribes. Because pairwise Procrustes distances between the two jaws differed substantially due to the different number of landmarks used for each jaw, we normalized each distance matrix (within each jaw type) for better visualization (0 = zero distance; 1 = maximal observed Procrustes distance between any pair of species within jaw type).

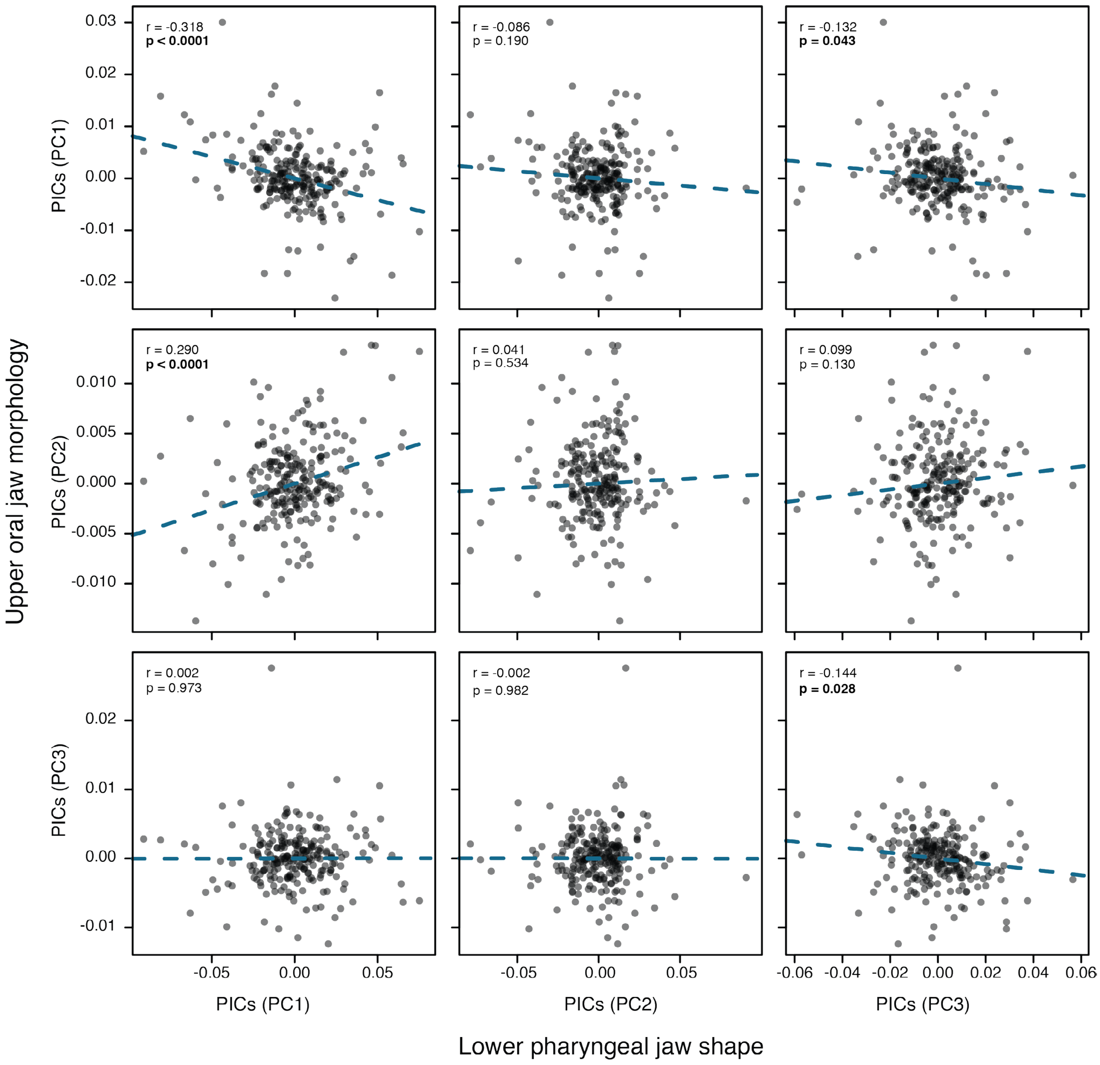

Figure S6. Evolutionary correlations of the first three PC-axes of oral jaw morphology (y-axes) and lower pharyngeal jaw shape (x-axes). Data points are phylogenetically independent contrasts of species means. Only the evolutionary correlation of PC1 and PC2 of oral jaw morphology with PC1 of lower pharyngeal jaw shape were stronger than expected for uncorrelated traits that evolved under Brownian motion (r = -0.21 – 0.23).

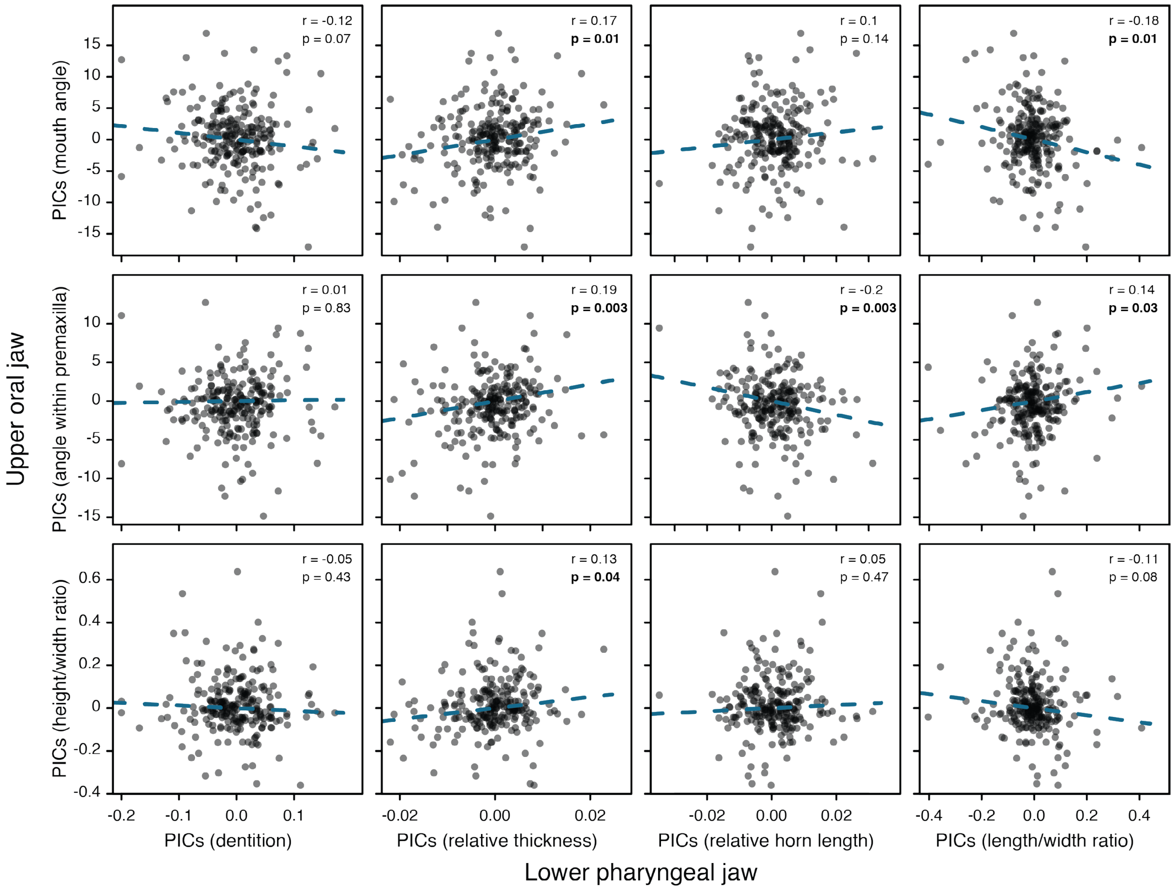

Figure S7. Evolutionary correlations of metric measurements of oral (y-axes) and lower pharyngeal (x-axes) jaws. Data points are phylogenetically independent contrasts of species means. All evolutionary correlations between the jaws fell within the null distribution for uncorrelated traits simulated under Brownian motion (r = -0.21 – 0.23).

Table S1. Sample size information of the studied species.

Tribe	Species	$N_{oral jaw}$	$N_{pharyngeal jaw}$
Bathybatini	*Bathybates fasciatus*	8	5
Bathybatini	*Bathybates ferox*	10	5
Bathybatini	*Bathybates graueri*	10	5
Bathybatini	*Bathybates hornii*	1	1
Bathybatini	*Bathybates leo*	7	5
Bathybatini	*Bathybates minor*	10	5
Bathybatini	*Bathybates vittatus*	5	5
Bathybatini	*Hemibates stenosoma*	9	5
Bathybatini	*Hemibates koningsi*	2	2
Benthochromini	*Benthochromis* sp. "horii mahale"	10	5
Benthochromini	*Benthochromis horii*	10	5
Benthochromini	*Benthochromis melanoides*	10	5
Benthochromini	*Benthochromis tricoti*	10	5
Boulengerochromini	*Boulengerochromis microlepis*	10	5
Cyphotilapiini	*Cyphotilapia* sp. "5-bar frontosa"	10	5
Cyphotilapiini	*Cyphotilapia frontosa*	10	5
Cyphotilapiini	*Cyphotilapia gibberosa*	10	5
Cyphotilapiini	*Ctenochromis benthicola*	6	5
Cyprichromini	*Cyprichromis coloratus*	10	5
Cyprichromini	*Cyprichromis* sp. "dwarf jumbo"	10	5
Cyprichromini	*Cyprichromis* sp. "jumbo"	10	5
Cyprichromini	*Cyprichromis leptosoma*	10	5
Cyprichromini	*Cyprichromis microlepidotus*	10	5
Cyprichromini	*Cyprichromis pavo*	10	5
Cyprichromini	*Cyprichromis zonatus*	10	5
Cyprichromini	*Paracyprichromis* sp. "brieni south"	10	5
Cyprichromini	*Paracyprichromis brieni*	10	5
Cyprichromini	*Paracyprichromis nigripinnis*	10	5
Ectodini	*Asprotilapia leptura*	10	5
Ectodini	*Aulonocranus dewindti*	10	5
Ectodini	*Callochromis macrops*	10	5
Ectodini	*Callochromis melanostigma*	10	5
Ectodini	*Callochromis pleurospilus*	10	5
Ectodini	*Cardiopharynx schoutedeni*	10	5
Ectodini	*Cunningtonia longiventralis*	10	5
Ectodini	*Cyathopharynx foae*	10	5
Ectodini	*Cyathopharynx furcifer*	10	5
Ectodini	*Ectodus descampsii*	10	5
Ectodini	*Ectodus* sp. "north"	10	5
Ectodini	*Enantiopus melanogenys*	10	5
Ectodini	*Grammatotria lemairii*	9	5
Ectodini	*Lestradea perspicax*	10	5
Ectodini	*Lestradea stappersii*	10	5
Ectodini	*Microdontochromis rotundiventralis*	10	5
Ectodini	*Microdontochromis tenuidentata*	10	5
Ectodini	*Ophthalmotilapia boops*	10	5
Ectodini	*Ophthalmotilapia nasuta*	10	5
Ectodini	*Ophthalmotilapia* sp. "paranasuta"	10	5
Ectodini	*Ophthalmotilapia ventralis*	10	5
Ectodini	*Ophthalmotilapia* sp. "white cap"	9	5
Ectodini	*Xenotilapia bathyphilus*	10	5
Ectodini	*Xenotilapia boulengeri*	10	5
Ectodini	*Xenotilapia caudafasciata*	9	5
Ectodini	*Xenotilapia flavipinnis*	10	5
Ectodini	*Xenotilapia longispinis*	10	5
Ectodini	*Xenotilapia nasus*	10	5
Ectodini	*Xenotilapia nigrolabiata*	10	5
Ectodini	*Xenotilapia ochrogenys*	10	5
Ectodini	*Xenotilapia ornatipinnis*	10	5
Ectodini	*Xenotilapia papilio*	4	5
Ectodini	*Xenotilapia papilio* (Katete)	10	5
Ectodini	*Xenotilapia sima*	10	5
Ectodini	*Xenotilapia singularis*	10	5
Ectodini	*Xenotilapia spilopterus*	10	5
Ectodini	*Xenotilapia* sp. "spilopterus north"	10	5
Ectodini	*Xenotilapia* sp. "papilio sunflower"	10	5
Eretmodini	*Eretmodus cyanostictus*	10	5
Eretmodini	*Eretmodus marksmithi*	10	5
Eretmodini	*Spathodus erythrodon*	10	5
Eretmodini	*Spathodus marlieri*	10	5
Eretmodini	*Tanganicodus irsacae*	10	5
Lamprologini	*Altolamprologus calvus*	10	5
Lamprologini	*Altolamprologus compressiceps*	10	5
Lamprologini	*Neolamprologus fasciatus*	10	5
Lamprologini	*Altolamprologus* sp. "compressiceps shell"	10	5
Lamprologini	*Chalinochromis* sp. "bifrenatus"	10	5
Lamprologini	*Chalinochromis brichardi*	10	5
Lamprologini	*Chalinochromis cyanophleps*	10	5
Lamprologini	*Chalinochromis* sp. "ndobhoi"	10	5

Table S1 (continued)

Tribe	Species	$N_{oral\ jaw}$	$N_{pharyngeal\ jaw}$
Lamprologini	*Julidochromis dickfeldi*	10	5
Lamprologini	*Julidochromis* sp. "kombe"	10	5
Lamprologini	*Julidochromis marlieri*	10	5
Lamprologini	*Julidochromis* sp. "marlieri south"	10	5
Lamprologini	*Julidochromis marksmithi*	9	5
Lamprologini	*Julidochromis ornatus*	10	5
Lamprologini	*Julidochromis* sp. "regani south"	10	5
Lamprologini	*Julidochromis regani*	10	5
Lamprologini	*Julidochromis* sp. "unterfels"	5	5
Lamprologini	*Lamprologus callipterus*	10	5
Lamprologini	*Lamprologus kungweensis*	10	5
Lamprologini	*Lamprologus lemairii*	10	5
Lamprologini	*Lamprologus meleagris*	8	5
Lamprologini	*Lamprologus ocellatus*	9	5
Lamprologini	*Lamprologus* sp. "ornatipinnis congo"	5	5
Lamprologini	*Lamprologus ornatipinnis*	9	5
Lamprologini	*Lamprologus* sp. "ornatipinnis zambia"	9	5
Lamprologini	*Lamprologus signatus*	10	5
Lamprologini	*Lamprologus speciosus*	10	5
Lamprologini	*Lepidiolamprologus attenuatus*	10	5
Lamprologini	*Lepidiolamprologus cunningtoni*	10	5
Lamprologini	*Lepidiolamprologus elongatus*	10	5
Lamprologini	*Lepidiolamprologus kamambae*	10	5
Lamprologini	*Lepidiolamprologus kendalli*	10	5
Lamprologini	*Lepidiolamprologus* sp. "meeli kipili"	7	5
Lamprologini	*Lepidiolamprologus mimicus*	10	5
Lamprologini	*Lepidiolamprologus profundicola*	8	5
Lamprologini	*Neolamprologus bifasciatus*	10	5
Lamprologini	*Neolamprologus boulengeri*	10	5
Lamprologini	*Neolamprologus brevis*	9	5
Lamprologini	*Neolamprologus brichardi*	10	5
Lamprologini	*Neolamprologus* sp. "brevis magara"	10	5
Lamprologini	*Neolamprologus buescheri*	10	5
Lamprologini	*Neolamprologus* sp. "caudopunctatus kipili"	10	5
Lamprologini	*Neolamprologus calliurus*	10	5
Lamprologini	*Neolamprologus caudopunctatus*	10	5
Lamprologini	*Neolamprologus chitamwebwai*	10	5
Lamprologini	*Neolamprologus christyi*	10	5
Lamprologini	*Neolamprologus crassus*	10	5
Lamprologini	*Neolamprologus* sp. "cygnus"	10	5
Lamprologini	*Neolamprologus cylindricus*	10	5
Lamprologini	*Neolamprologus* sp. "eseki"	10	5
Lamprologini	*Neolamprologus falcicula*	10	5
Lamprologini	*Neolamprologus* sp. "falcicula mahale"	11	5
Lamprologini	*Neolamprologus furcifer*	10	5
Lamprologini	*Neolamprologus* sp. "furcifer ulwile"	10	5
Lamprologini	*Neolamprologus gracilis*	5	5
Lamprologini	*Neolamprologus* sp. "gracilis tanzania"	10	5
Lamprologini	*Neolamprologus helianthus*	5	5
Lamprologini	*Neolamprologus* sp. "kombe"	10	5
Lamprologini	*Lamprologus laparogramma*	9	5
Lamprologini	*Neolamprologus leleupi*	3	5
Lamprologini	*Neolamprologus longior*	10	5
Lamprologini	*Neolamprologus leloupi*	10	5
Lamprologini	*Neolamprologus marunguensis*	5	5
Lamprologini	*Neolamprologus meeli*	10	5
Lamprologini	*Neolamprologus modestus*	10	5
Lamprologini	*Neolamprologus mondabu*	10	5
Lamprologini	*Neolamprologus multifasciatus*	10	5
Lamprologini	*Neolamprologus mustax*	10	5
Lamprologini	*Neolamprologus niger*	10	5
Lamprologini	*Neolamprologus nigriventris*	5	5
Lamprologini	*Neolamprologus obscurus*	10	5
Lamprologini	*Neolamprologus olivaceous*	6	5
Lamprologini	*Neolamprologus pectoralis*	6	5
Lamprologini	*Neolamprologus petricola*	10	5
Lamprologini	*Neolamprologus pleuromaculatus*	4	4
Lamprologini	*Neolamprologus prochilus*	10	5
Lamprologini	*Neolamprologus pulcher*	10	5
Lamprologini	*Neolamprologus savoryi*	10	5
Lamprologini	*Neolamprologus sexfasciatus*	10	5
Lamprologini	*Neolamprologus similis*	10	5
Lamprologini	*Neolamprologus splendens*	5	5
Lamprologini	*Neolamprologus tetracanthus*	10	5
Lamprologini	*Neolamprologus timidus*	10	5
Lamprologini	*Neolamprologus toae*	10	5
Lamprologini	*Neolamprologus tretocephalus*	10	5
Lamprologini	*Neolamprologus ventralis* (Burundi)	6	5
Lamprologini	*Neolamprologus* sp. "ventralis stripe"	7	5

Table S1 (continued)

Tribe	Species	$N_{oral jaw}$	$N_{pharyngeal jaw}$
Lamprologini	*Neolamprologus walteri*	9	5
Lamprologini	*Telmatochromis bifrenatus*	10	5
Lamprologini	*Telmatochromis brachygnathus*	9	5
Lamprologini	*Telmatochromis brichardi*	10	5
Lamprologini	*Telmatochromis* sp. "dhonti north"	9	5
Lamprologini	*Telmatochromis dhonti*	10	5
Lamprologini	*Telmatochromis* sp. "dhonti twiyu"	10	5
Lamprologini	*Telmatochromis* sp. "longola"	1	1
Lamprologini	*Telmatochromis* sp. "shell"	10	5
Lamprologini	*Telmatochromis temporalis*	10	5
Lamprologini	*Telmatochromis vittatus*	10	5
Lamprologini	*Variabilichromis moorii*	10	5
Limnochromini	*Baileychromis centropomoides*	9	5
Limnochromini	*Gnathochromis permaxillaris*	10	5
Limnochromini	*Limnochromis abeelei*	10	5
Limnochromini	*Greenwoodochromis bellcrossi*	10	5
Limnochromini	*Greenwoodochromis christyi*	10	5
Limnochromini	*Limnochromis staneri*	10	5
Limnochromini	*Limnochromis auritus*	10	5
Limnochromini	*Reganochromis calliurus*	10	5
Limnochromini	*Tangachromis dhanisi*	3	3
Limnochromini	*Triglachromis otostigma*	10	5
Perissodini	*Haplotaxodon microlepis*	7	5
Perissodini	*Perissodus eccentricus*	9	5
Perissodini	*Perissodus microlepis*	10	5
Perissodini	*Plecodus elaviae*	9	5
Perissodini	*Plecodus multidentatus*	8	5
Perissodini	*Plecodus paradoxus*	10	5
Perissodini	*Plecodus straeleni*	10	5
Perissodini	*Xenochromis hecqui*	9	5
Trematocarini	*Trematocara caparti*	3	3
Trematocarini	*Trematocara macrostoma*	10	5
Trematocarini	*Trematocara marginatum*	10	5
Trematocarini	*Trematocara nigrifrons*	10	5
Trematocarini	*Trematocara stigmaticum*	6	5
Trematocarini	*Trematocara unimaculatum*	9	5
Trematocarini	*Trematocara zebra*	10	5
Tropheini	*Ctenochromis horei*	10	5
Tropheini	*Gnathochromis pfefferi*	10	5
Tropheini	*Interochromis loocki*	10	5
Tropheini	*Limnotilapia dardennii*	9	5
Tropheini	*Lobochilotes labiatus*	9	5
Tropheini	*Petrochromis ephippium*	9	5
Tropheini	*Petrochromis famula*	10	5
Tropheini	*Petrochromis fasciolatus*	10	5
Tropheini	*Petrochromis* sp. "giant"	3	5
Tropheini	*Petrochromis horii*	10	5
Tropheini	*Petrochromis* sp. "orthognathus ikola"	10	5
Tropheini	*Petrochromis* sp. "kazumba"	10	5
Tropheini	*Petrochromis* sp. "kipili brown"	10	5
Tropheini	*Petrochromis macrognathus*	10	5
Tropheini	*Petrochromis* sp. "moshi yellow"	10	5
Tropheini	*Petrochromis orthognathus*	10	5
Tropheini	*Petrochromis polyodon*	9	5
Tropheini	*Petrochromis* sp. "macrognathus rainbow"	10	5
Tropheini	*Petrochromis* sp. "red"	10	5
Tropheini	*Petrochromis* sp. "polyodon texas"	10	5
Tropheini	*Petrochromis trewavasae*	10	5
Tropheini	*Pseudosimochromis curvifrons*	10	5
Tropheini	*Pseudosimochromis babaulti*	10	5
Tropheini	*Simochromis diagramma*	10	5
Tropheini	*Pseudosimochromis marginatus*	10	5
Tropheini	*Pseudosimochromis marginatus* (North)	9	5
Tropheini	*Pseudosimochromis babaulti* (South)	9	5
Tropheini	*Tropheus brichardi*	10	5
Tropheini	*Tropheus* sp. "brichardi kipili"	10	5
Tropheini	*Tropheus duboisi*	10	5
Tropheini	*Tropheus* sp. "kirschfleck"	10	5
Tropheini	*Tropheus* sp. "lukuga"	10	5
Tropheini	*Tropheus* sp. "lunatus"	10	5
Tropheini	*Tropheus moorii*	10	5
Tropheini	*Tropheus* sp. "murago"	10	5
Tropheini	*Tropheus* sp. "mpimbwe"	10	5
Tropheini	*Tropheus* sp. "black"	10	5
Tropheini	*Tropheus polli*	10	5
Tropheini	*Tropheus* sp. "red"	10	5
12 tribes	234 taxa	2171	1154
